# Corticolimbic connectivity mediates the relationship between pubertal timing and mental health problems

**DOI:** 10.1017/S0033291723001472

**Published:** 2023-12

**Authors:** Nandita Vijayakumar, Sarah Whittle, Timothy J. Silk

**Affiliations:** 1 Deakin University, Centre for Social and Early Emotional Development, School of Psychology, Faculty of Health, Geelong, Victoria, Australia; 2 Centre for Adolescent Health, Murdoch Children's Research Institute, Parkville, Victoria, Australia; 3 Department of Psychiatry, Melbourne Neuropsychiatry Centre, The University of Melbourne and Melbourne Health, Melbourne, Victoria, Australia; 4 Developmental Imaging, Murdoch Children's Research Institute, Parkville, Victoria, Australia

**Keywords:** brain connectivity, family environment, mental health, pubertal timing, resting-state

## Abstract

**Background:**

Undergoing puberty ahead of peers (‘earlier pubertal timing’) is an important risk factor for mental health problems during early adolescence. The current study examined pathways between pubertal timing and mental health via connectivity of neural systems implicated in emotional reactivity and regulation (specifically corticolimbic connections) in 9- to 14-year-olds.

**Method:**

Research questions were examined in the Adolescent Brain Cognitive Development (ABCD) Study, a large population representative sample in the United States. Linear mixed models examined associations between pubertal timing and resting-state corticolimbic connectivity. Significant connections were examined as potential mediators of the relationship between pubertal timing and mental health (withdrawn depressed and rule-breaking) problems. Exploratory analyses interrogated whether the family environment moderated neural risk patterns in those undergoing puberty earlier than their peers.

**Results:**

Earlier pubertal timing was related to decreased connectivity between limbic structures (bilateral amygdala and right hippocampus) and the cingulo-opercular network, left amygdala and somatomotor (mouth) network, as well as between the left hippocampus and ventral attention network and visual network. Corticolimbic connections also mediated the relationship between earlier pubertal timing and increased withdrawn depressed problems (but not rule-breaking problems). Finally, parental acceptance buffered against connectivity patterns that were implicated in withdrawn depressed problems in those undergoing puberty earlier than their peers.

**Conclusion:**

Findings highlight the role of decreased corticolimbic connectivity in mediating pathways between earlier pubertal timing and withdrawn depressed problems, and we present preliminary evidence that the family environment may buffer against these neural risk patterns during early adolescence.

Undergoing pubertal maturation earlier than one's peers (referred to as earlier pubertal timing) is consistently identified as a predictor for adolescent mental health problems (Ullsperger & Nikolas, 2017). Adolescence is also characterized by critical development of the brain's functional networks, with pubertal processes driving certain aspects of maturation (Vijayakumar, Op de Macks, Shirtcliff, & Pfeifer, 2018). In particular, it is often postulated that puberty may play a role in changing connectivity between cortical and limbic structures (Colich & McLaughlin, 2022). Corticolimbic functional connectivity is also commonly implicated in mental health problems (Chahal, Gotlib, & Guyer, 2020; Marusak et al., 2016; Schmitt, 2022), and is thought to directly contribute to alterations in emotion regulation. It thus represents a potential pathway between earlier pubertal timing and mental health problems. Understanding this mechanistic pathway will aid the identification of targets for mental health interventions during the adolescent years.

## Puberty and corticolimbic connectivity

During puberty, the release of androgen and gonadal hormones trigger the process of physical and sexual development. These same hormones also act via androgen and estrogen receptors in the brain to modulate neurotransmission and influence synaptic function, myelination, and neurite growth (Piekarski et al., 2017). Hormone receptor density is highest within limbic regions, particularly the amygdala and hippocampus (Kashon & Sisk, 1994; Milner et al., 2010). While receptor density is more sparse in the cortex, recent rodent studies illustrate that pubertal processes also influence cortical development – particularly within the medial PFC (Delevich, Klinger, Okada, & Wilbrecht, 2021). Further, the properties of neuronal projections between the amygdala and PFC are also in flux during puberty (Cressman et al., 2010; Cunningham, Bhattacharyya, & Benes, 2002), suggesting that corticolimbic connections are changing.

While a growing literature examines maturation of the human brain during puberty, few studies have focused on connectivity. Those directly interrogating corticolimbic connectivity have noted reductions during resting-state with increasing pubertal stage (conceptualized as the observable physical changes driven by hormones), such as decreased hippocampal-dorsal ACC connectivity in females (van Duijvenvoorde, Westhoff, de Vos, Wierenga, & Crone, 2019). Similarly, recent investigations in a large representative US sample have reported decreased connectivity between the amygdala and cingulo-opercular network (CON; comprising the dorsal anterior cingulate and insular cortices) at higher pubertal stage (Thijssen, Collins, & Luciana, 2020). Others have examined task-evoked connectivity, with some identifying similar decreases in amygdala-insula connectivity with increasing androgen hormones [specifically dehydroepiandrosterone (DHEA) in females (Barendse et al., 2020)] and longitudinal decreases in amygdala-OFC connectivity with increasing testosterone levels during threat-processing (Spielberg et al., 2015). However, inconsistences include increased amygdala-dorsal ACC connectivity with testosterone levels during threat-processing (in males, Barendse et al., 2020) and increased amygdala-vmPFC connectivity with faster pubertal tempo (i.e. longitudinal increases in pubertal stage) during emotion processing (Miller et al., 2020). One recent study also failed to identify any associations between connectivity during emotion processing and pubertal timing (Colich et al., 2023). Taken together, there is generally support for corticolimbic connectivity – particularly between the amygdala and prefrontal cortex – changing during puberty. However, with the exception of two studies (Barendse et al., 2020; Colich et al., 2023), most have not specifically interrogated pubertal timing – the stage of an individual relative to same-age peers. While studies use varying methods of modeling age, it is unclear whether they are capturing individual differences in timing. Therefore, it remains uncertain whether connectivity patterns reflect normative changes through the progression of pubertal stages, or accelerated patterns (beyond the group average) in those with early timing.

## Implications for mental health

Notably, aberrant patterns of corticolimbic connectivity are consistently implicated in mental health problems (Chahal et al., 2020; Marusak et al., 2016; Schmitt, 2022). Many studies have identified altered resting-state amygdala-PFC connectivity in adolescent depression; several highlight decreased connectivity with higher-order cognitive regions (Scheuer et al., 2017; Tang et al., 2018; Wu et al., 2020), but there remain inconsistencies (Brieant, Sisk, & Gee, 2021). While less research has interrogated externalizing problems, some have implicated similar corticolimbic networks (Kim et al., 2016; Thijssen, Collins, Weiss, & Luciana, 2021). Critically, accelerated reductions in amygdala-PFC connectivity is thought to reflect dysregulated top-down control of limbic reactivity to environmental stressors, thus suggesting aberrant emotion processing and regulation (Pannekoek et al., 2014; Perlman et al., 2012). Others propose that accelerated maturation may prematurely terminate sensitive periods of neural plasticity that support the acquisition of emotion regulation skills (Callaghan & Tottenham, 2016). Meta-analyses have also implicated increased connectivity between the amygdala and default-mode network in adolescent depression (Tang et al., 2018), which is thought to reflect recursive self-referential thoughts and negative affective response patterns (Cooney, Joormann, Eugène, Dennis, & Gotlib, 2010; Cullen et al., 2014). However, some note decreased amygdala connectivity when considering the vmPFC alone – one component of the default mode network (Hanson et al., 2019). Thus, although inconsistencies remain with regards to directionality, findings suggest that altered resting-state corticolimbic connectivity may represent a potential pathway between pubertal timing and mental health problems.

Indeed, prominent models of neurodevelopment propose that pubertal processes drive the maturation of limbic systems early in adolescence, while experiential factors lead to protracted maturation of cortical networks into young adulthood, thus resulting in ‘normative’ patterns of corticolimbic mismatch (i.e. relatively earlier limbic compared to cortical maturation) (Shulman et al., 2016). Accelerated maturation in individuals undergoing puberty earlier than their peers may therefore exacerbate this mismatch, with resultant corticolimbic dysregulation increasing risk for mental health problems. However, few have investigated pathways between pubertal timing, corticolimbic connectivity and mental health. Kircanski et al. (2019) showed that accelerated frontolimbic white matter development during early puberty predicted greater depression symptomatology. Spielberg et al. (2015) showed that increased testosterone was related to decreased amygdala-OFC functional connectivity during threat processing, which was in turn related to increased withdrawn temperament. Barendse et al. (2020) found similar patterns predicting anxiety in adolescent females, but opposing effects in males. To our knowledge, the only study to examine resting-state connectivity failed to identify any associations between puberty-related network changes and internalizing problems, although it did not focus on corticolimbic connections (Ernst et al., 2019).

*Contextual amplification* models of puberty propose that early timing may be considered a biological marker of vulnerability that interacts with environmental stressors to predict mental health problems (Ge & Natsuaki, 2009). For example, some research suggests that early timing is only related to depression and conduct problems in the context of adverse family environments (Rudolph & Troop-Gordon, 2010). Further, Vijayakumar et al. (2022) showed that supportive environments act to buffer against associations between timing and depressive symptoms and rule-breaking behavior problems. While unfavorable social environments may exacerbate the stress of undergoing puberty earlier than peers, with multiple concurrent challenges overtaxing under-developed cognitive and emotional resources and leading to mental health problems, favorable social contexts may better support individuals to navigate through the challenges of puberty without adverse consequences for their mental health. Thus, social contexts may play a role in moderating corticolimbic connectivity that is implicated in mental health problems among those experiencing puberty earlier than peers. A growing literature highlights the potential for social contexts – particularly family environments (Pozzi et al., 2021; Thijssen et al., 2020, 2017) – to influence connectivity, and there is preliminary evidence that parenting may moderate associations between pubertal timing and vlPFC function during threat processing (Barbosa et al., 2018). Identifying such social contexts can help us identify those at heightened vulnerability during the early adolescent years.

## The current study

The goal of the current study was to investigate associations between pubertal timing, corticolimbic connectivity and mental health problems. We focus specifically on (i) connectivity of two limbic structures, the amygdala and hippocampus, given the density of pubertal hormone receptors in these regions, and (ii) depressive and rule-breaking problems as outcome measures as they have been most consistently implicated in relation to earlier pubertal timing (Vijayakumar & Whittle, 2023). We examined their connectivity to large scale, distributed functional networks in the cortex during resting-state, building upon research that has implicated these networks in mental health problems. Associations were investigated in the Adolescent Brain Cognitive Development (ABCD) Study, a large population representative sample in the United States. The first aim was to examine associations between pubertal timing and corticolimbic connectivity, hypothesizing that earlier timing would be related to decreased connectivity between amygdala/hippocampus and higher-order cognitive networks (e.g. cingulo-opercular and frontoparietal networks). Next, we examined whether functional connectivity mediated the well-characterized association between pubertal timing and mental health problems. It was hypothesized that decreased connectivity of the amygdala/hippocampus and higher-order networks would significantly mediate the association between earlier timing and greater mental health problems. Finally, an exploratory aim was to identify family environments that moderate the functional connectivity patterns implicated in mental health problems. We hypothesized that positive family environments (characterized by either high levels of parental acceptance or low levels of family conflict) would buffer patterns of functional connectivity that were related to mental health problems (e.g. positive family environments would lead to less reductions in connectivity in adolescents with early timing) while negative family environments would exacerbate such connectivity patterns.

## Methods

The sample was derived from the ABCD Study (Release 4.0 downloaded from the National Institute of Mental Health Data Archive's ABCD Collection). Analyses focused on data (for puberty, rsfMRI, mental health and the family environment) from baseline and 2-year follow-up assessments when participants were 9–11 and 10.5–13.5 years old, respectively. Data points were excluded in the following successive order: missing puberty (*N* = 602), mental health (*N* = 2243) or rsfMRI data (*N* = 1659), as well as rsfMRI data recommended for exclusion (described further in below, *N* = 2310). This resulted in a final sample of 15524 data points (7557 females) from 10501 participants (5143 females), including 8884 unique families (and 1571 sibling sets). Data was collected across 22 sites in the United States. Statistical modeling focused on between-subject effects in this dataset that was nested within participant, family, and site.

Ethical review and approval of ABCD's protocol, as well as informed consent procedures, are outlined by Clark et al. (2017). The use of pre-existing and non-identifiable data in the current analyses was approved by Deakin University's Human Research Ethics Committee.

### Measures

#### Pubertal timing

Adolescents completed the Pubertal Development Scale (PDS, Petersen, Crockett, Richards, & Boxer, 1988) consisting of five questions that assess height growth, body hair and skin changes, as well as breast development and menarche in females, and facial hair and voice changes in males. Items were rated on a 4-point scale and mean scores were calculated across items. Using data from baseline and 2-year follow-up assessments, pubertal timing was calculated within each sex by regressing age from pubertal stage using a linear mixed model: PDS ~ age + age^2^ + (1|subject_id) + (1|family_id) + (1| site_id). A quadratic (fixed) effect of age was modeled given nonlinear trends identified in prior literature (Vijayakumar et al., 2021; Wierenga et al., 2018), and random effects of subject, family and site IDs accounted for the nested nature of the data. The subject-specific random effect and the model residuals were summed as a measure of pubertal timing (i.e. pubertal stage relative to same-age and -sex peers), reflecting ‘stable’ intercept differences per individual (across time points) in addition to residual differences at each time point. This allowed us to estimate a unique value for pubertal timing for each time point (for participants who contributed data from two waves). Positive and negative values reflect earlier and later pubertal timing, respectively. Refer to online Supplementary Fig. S1 for an illustration of the association between pubertal stage and age, and online Supplementary Fig. S2 for the distribution of pubertal timing values.

#### rsfMRI

Adolescents underwent MRI scans in a 3 T scanner (Siemens, Philips, or General Electric). They completed four or five resting-state MRI scans (each lasting 5 min), with their eyes open and fixated on a crosshair (further details provided by Casey et al., 2018). Pre-processing was undertaken by the ABCD Data Analysis and Informatics Core using a standardized pipeline. Pre-processing involved removal of initial frames, normalization, regression and temporal filtering (Hagler et al., 2019). Time courses were then projected onto each participant's cortical surface, and average cortical time courses were calculated for 12 resting state networks defined by the Gordon parcellation scheme [auditory network (AN), cingulo-opercular network (CON), cinguloparietal network (CPN), dorsal attention network (DAN), default mode network (DMN), frontoparietal network (FPN), retrosplenial temporal network (RTN), sensorimotor (hand) network (SMN-H), sensorimotor (mouth) network (SMN-M), salience network (SN), ventral attention network (VAN) and visual network (VN)]. Average time courses were also calculated for FreeSurfer-defined amygdala and hippocampus (separately for each hemisphere). Average time courses were calculated across available runs, with runs of fewer than 100 usable time points (out of 375 acquired) excluded from the average. Correlations between limbic ROIs and cortical networks were calculated and Fisher-*Z* transformed, providing summary measures of cortico-limbic connectivity. The current study utilized these indices of connectivity via the ABCD Study's tabulated data. As recommended by ABCD Release Notes 4.0, data from the tabulated output was only included based upon the recommended inclusion flag for rsfMRI data (i.e. excluded if ‘imgincl_rsfmri_include = 0’). Two additional motion metrics were included as covariates in analyses: mean framewise displacement (motion) and outlier count (rsfmri_c_ngd_nvols – rsfmri_c_ngd_ntpoints).

#### Mental health problems

Parents completed the Child Behavior Checklist for Ages 6–18 (CBCL/6–18). Analyses focused on withdrawn depression and rule-breaking syndrome raw scores. To deal with positive skew, Poisson distribution was used in statistical modeling.

#### Family environment

Adolescents completed the Children's Report of Parental Behavior Inventory (CRPBI) and Family Environment Scale (FES). CRPBI includes a parental acceptance subscale comprising 5 items (rated on a 3-point Likert Scale), which was completed about the primary caregiver. Of note, this measure was completed during the baseline and 1-year follow-up assessments, and thus there is an approximately 1-year time-lag with other variables of interest for a subset of datapoints (i.e. those relating to 2-year follow-up). FES includes a subscale on family conflict comprising 9 items about members of the family unit (e.g. ‘family members often criticize each other’). For both scales, items are summed, with higher scores reflecting greater parental acceptance and family conflict, respectively.

### Statistical analyses

For all statistical analyses, continuous predictors were standardized prior to running models. All models accounted for the nested nature of the dataset (within participant, family and site).

#### Puberty and corticolimbic connectivity

Linear mixed models examined whether corticolimbic connectivity differed across pubertal timing (i.e. connectivity ~ timing), using *lme4* (Bates, Mächler, Bolker, & Walker, 2015) in R. While pubertal stage was not a focus of this study, we also ran models that examined associations between connectivity and pubertal stage (i.e. PDS score) to aide interpretation of findings related to pubertal timing. Separate models were conducted for each connectivity variable (*N* = 48). Covariates included the fixed effects of sex, mean framewise displacement, outlier count, and participation following the start of the COVID19 pandemic (yes/no). Random effects of subject, family and site IDs accounted for the nested nature of the data. To ensure that findings were robust, we undertook a within sample split-half replication; our sample was split into discovery and replication datasets [based on family and subject clustering to ensure independence, using ‘groupKfold’ in *caret* (Kuhn, 2008)], and mixed models were conducted on each dataset. We controlled for multiple comparisons at a false discovery rate (FDR) of 5% in each of the discovery and replication dataset, and only findings surviving FDR correction in both datasets were considered significant.

Supplemental analyses examined associations between corticolimbic connectivity and pubertal timing when incorporating additional covariates of income-to-needs ratio (INR; calculation outlined in the Supplement) and race/ethnicity. These variables were not included in primary models to avoid further loss of sample size due to missingness [10% of the sample (*n* = 1450) were missing one or more variables]. We also explored whether associations between corticolimbic connectivity and pubertal timing differed across sex (connectivity ~ timing × sex) and age (connectivity ~ timing × age) but failed to identify any significant effects (see online Supplementary Table S3).

#### Pubertal timing, corticolimbic connectivity, and mental health

For significant connections, we examined potential mediation of the association between timing and mental health problems via corticolimbic connectivity. We undertook mediation analyses through Bayesian modeling with *brms* (Bürkner, 2017) which supports mediation with multilevel models with multiple random effects. Modeling involved calculation of 95% credible intervals (CIs) to gauge uncertainty of indirect estimates, using Markov chain Monte Carlo simulations. Given the observed data, there is 95% probability of identifying the true estimate within the credible interval. We calculated CIs based on highest density intervals, which always include the mode of posterior distributions. As we did not have prior knowledge of anticipated mediation effects, we used weakly informative priors that decrease the likelihood of estimating unrealistically small or large effects, without substantively influencing regression parameters (Gelman, Jakulin, Pittau, & Su, 2008). We used the default number of 4 Markov chains but increased sampling iterations to 10000 (with 2000 warmup) as chains did not adequately converge when using the default value of 2000.

Separate mediation models examined each combination of connectivity and mental health problems (withdrawn depression and rule-breaking). For each model, a multivariate framework specified both paths A and B, with the same random effects and covariates as the linear mixed effects models. Path A was modeled with the (default) Gaussian distribution, while Path B was modeled with a Poisson distribution due to non-normality of the CBCL variables. Model convergence was examined based on Rhat values (< 1.05) and posterior predictive checks. Next, the indirect effect was calculated using the product of coefficients method, by multiplying the regression coefficient for path A and B in the posterior distribution to derive a distribution for the mediation effect (similar to bootstrapped mediation). Likelihood of mediation was determined upon the 95% CI range (reflecting the highest density interval across posterior distributions). Code for multivariate models and calculation of indirect effects is presented in online Supplementary Material.

#### Moderation by the family environment

Exploratory models examined whether the family environment moderated associations between pubertal timing and corticolimbic connectivity. For connectivity variables identified as significant mediators, linear mixed models (with *lme4*) were used to examine whether parental acceptance or family conflict interacted with pubertal timing to predict corticolimbic connectivity (connectivity ~ pubertal timing × family environment), with the same covariates and random effects as prior models. Finally, moderated mediation models examined whether the family environment moderated indirect pathways between timing and mental health problems. Specifically, models for path A and C incorporated a main effect of family environment and an interaction term with pubertal timing (separately for parental acceptance and family conflict). Moderation effects were further interrogated by examination of mediation pathways at high ( + 1s.d. above mean) and low (−1s.d. below mean) levels of the family environment.

## Results

The sample comprised 52.9% White, 19.8% Hispanic, 14.7% Black/African American, and 12.2% Other/Mixed races. Refer to Tables 1 and 2 for further descriptive information. A correlation plot of primary variables is presented in online Supplementary Fig. S3.

**Table 1.** Sample characteristics

	Females								Males							
	Baseline				2-year follow-up				Baseline				2-year follow-up			
	Mean	s.d.	Min	Max	Mean	s.d.	Min	Max	Mean	s.d.	Min	Max	Mean	s.d.	Min	Max
Age	9.92	0.62	8.92	11.00	11.88	0.64	10.67	13.83	9.96	0.63	8.92	11.08	11.95	0.64	10.58	13.58
PDS	1.69	0.54	1.00	4.00	2.33	0.66	1.00	4.00	1.64	0.49	1.00	4.00	1.85	0.54	1.00	4.00
CBCL depression	4.99	5.51	0.00	51.00	5.13	5.80	0.00	38.00	4.91	5.44	0.00	49.00	4.68	5.44	0.00	50.00
CBCL delinquency	3.64	5.07	0.00	42.00	3.36	5.04	0.00	43.00	4.86	6.19	0.00	49.00	4.34	5.78	0.00	50.00
CRPBI	2.80	0.29	1.00	3.00	2.82	0.27	1.20	3.00	2.77	0.30	1.00	3.00	2.80	0.28	1.00	3.00
FES	4.04	2.88	0.05	15.39	4.11	2.77	0.05	14.78	4.04	2.82	0.03	15.39	4.22	2.80	0.06	14.78
INR	9.92	0.62	8.92	11.00	11.88	0.64	10.67	13.83	9.96	0.63	8.92	11.08	11.95	0.64	10.58	13.58

CBCL, Child Behavior Checklist; CRPBI, Children's Report of Parental Behavior Inventory; FES, Family Environment Scale; INR, Income-to-need ratio (calculation outlined in online Supplementary Material); PDS, Pubertal Development Scale.

**Table 2.** Race/ethnicity of the sample

	Females		Males	
	*N*	%	*N*	%
Black	780	15	745	14
Hispanic	1019	20	1045	20
White	2678	52	2915	54
Other	644	13	633	12
Missing	22	<1	20	<1

### Puberty and corticolimbic connectivity

**Figure 1.**
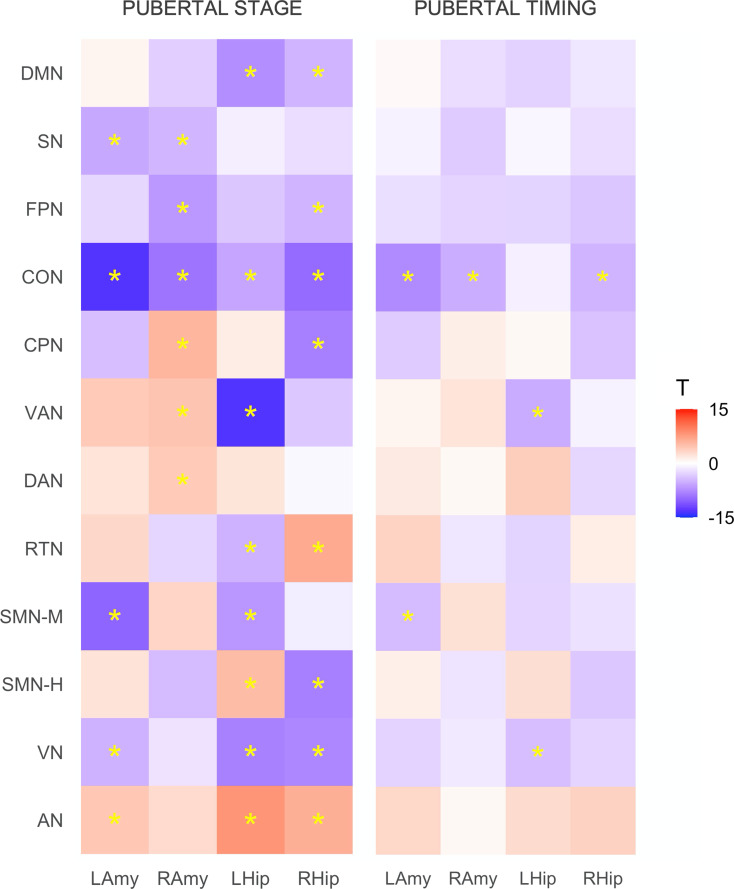
Associations between resting-state corticolimbic connectivity and (i) pubertal stage and (ii) pubertal timing. Heatmaps represent *T* statistics (*T*) of models on the full dataset, while significance (*) is based on FDR 0.05 for both discovery and replication sets. LAmy, Left Amygdala; RAmy, Right Amygdala; LHip, Left Hippocampus; RHip, Right Hippocampus; DMN, Default Mode Network; SN, Salience Network; FPN, Frontoparietal Network; CON, Cingulo-Opercular Network; CPN, Cingulo-Parietal Network; VAN, Ventral Attention Network; DAN, Dorsal Attention Network; RSN, Retrosplenial Temporal Network; SMN-M, Somatomotor Network – Mouth; SMN-H, Somatomotor Network – Hand; VN, Visual Network; AN, Auditory Network.

Patterns of resting-state corticolimbic connectivity across pubertal stage and timing are illustrated in Fig. 1 (with statistics presented in online Supplementary Table S2). More advanced pubertal stage was predominantly related to decreased connectivity across corticolimbic networks, with strongest effects present for left amygdala-CON and left hippocampus-VAN. Comparatively, increased connectivity was evident for limbic-AN and a few other connections. Earlier pubertal timing was associated with decreased functional connectivity between limbic structures (bilateral amygdala and right hippocampus) and CON, left amygdala and SMN-M, left hippocampus and VAN, and left hippocampus and VN. These associations remained significant when controlling for race/ethnicity and INR, aside from connectivity of the left hippocampus and VN (refer to Supplement).

### Pubertal timing, corticolimbic connectivity, and mental health

**Figure 2.**
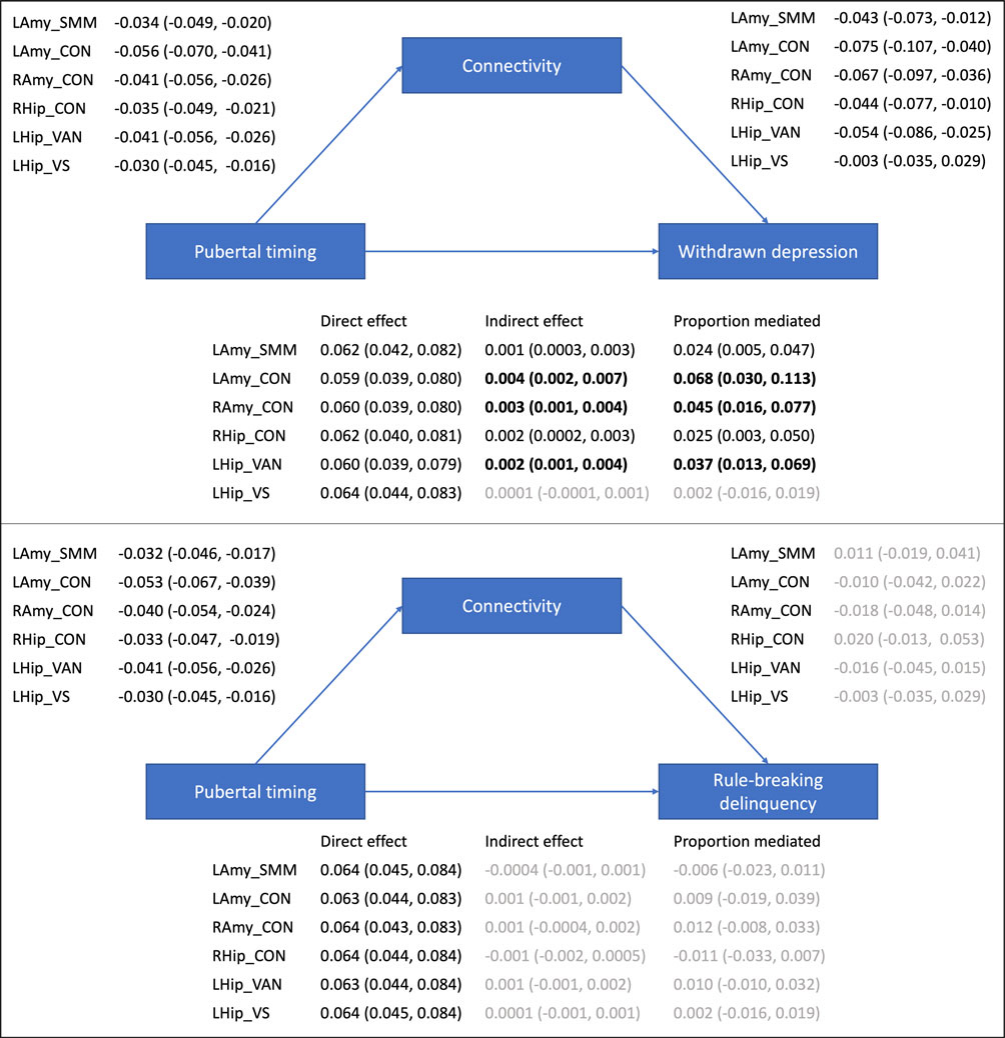
Mediation models examining indirect pathways between pubertal timing and mental health problems via resting-state corticolimbic connectivity (estimates and 95% CIs presented). LAmy, Left Amygdala; RAmy, Right Amygdala; LHip, Left Hippocampus; RHip, Right Hippocampus; CON, Cingulo-Opercular Network, Somatomotor Network – Mouth; VAN, Ventral Attention Network; VN, Visual Network.

Mediation analyses examined whether the six corticolimbic connections that were significantly related to pubertal timing mediated pathways to mental health problems (statistics from all multivariate models are presented in online Supplementary Table S4). As illustrated in Fig. 2, Bayesian analyses revealed a direct effect of pubertal timing on withdrawn depression, as well as an indirect effect mediated via five of six corticolimbic connections (accounting for up to 7% of the total effect). In other words, CIs revealed a 95% probability that the direct and indirect effects lie within intervals that do not contain zero. Specifically, decreased connectivity (of left amygdala-CON, right amygdala-CON, right hippocampus-CON, left hippocampus-VAN and left amygdala-SMN-M) mediated the relationship between earlier pubertal timing and higher levels of withdrawn depression. Of note, the most support was evident (at 99% credibility intervals) for bilateral amygdala-CON and left hippocampus-VAN connectivity, and there remained support for these three connections when controlling for race/ethnicity and INR (refer to Supplement). There was also a direct effect of pubertal timing on rule-breaking problems, with earlier timing predicting higher levels of delinquency. However there were no indirect pathways between timing and rule-breaking problems via corticolimbic connectivity.

### Pubertal timing, family environment, and corticolimbic connectivity

**Figure 3.**
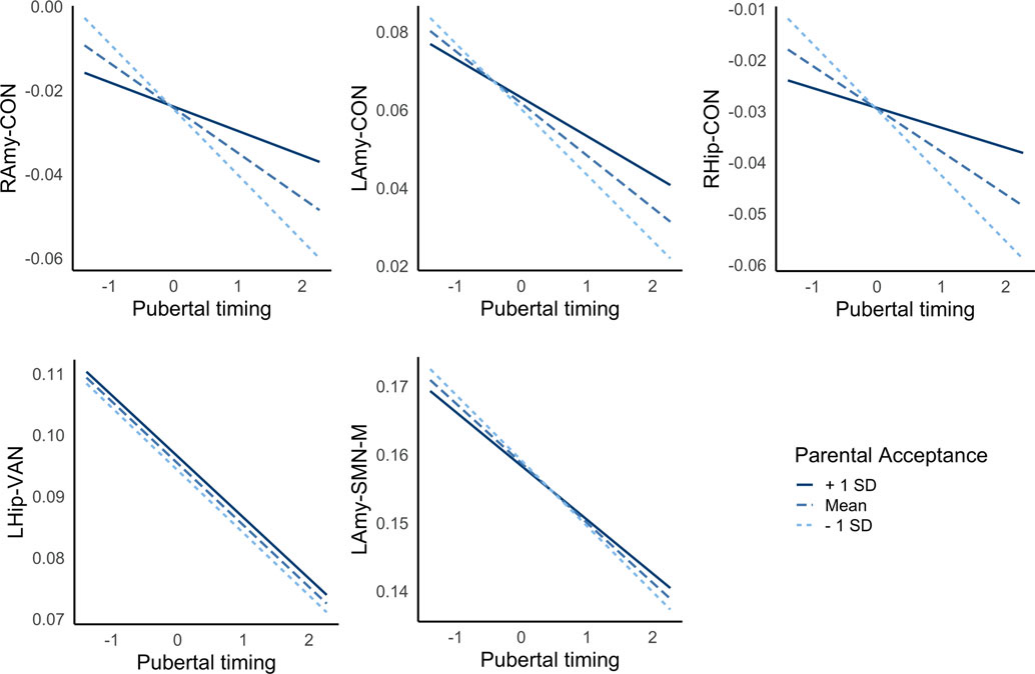
Interactions between pubertal timing and parental acceptance predict resting-state amygdala-CON connectivity. Positive and negative values reflect earlier and later pubertal timing, respectively. LAmy, Left Amygdala; RAmy, Right Amygdala; CON, Cingulo-Opercular Network; SMN-M, Somatomotor Network – Mouth; VAN, Ventral Attention Network.

Exploratory analyses failed to identify significant moderation between pubertal timing and family conflict in predicting connectivity, but significant moderation was identified for parental acceptance across left amygdala-CON, right amygdala-CON and right hippocampus-CON (but not left hippocampus-VAN or left-amygdala-SMN-M; see Table 3). As illustrated in Fig. 3, high levels of parental acceptance buffered against reductions in connectivity, while low levels of parental acceptance increased reductions in connectivity, in those with earlier timing. Further, Bayesian analyses revealed moderation of indirect pathways between pubertal timing and withdrawn depression by parental acceptance via left amygdala-CON [−0.001 (CIs −0.003 to −0.0002)] and right amygdala-CON [−0.001 (CIs −0.003 to −0.0002)], but not right hippocampus-CON [−0.001 (CIs −0.002 to 0.000)]. In other words, CIs revealed a 95% probability that moderation of the indirect pathways via amygdala-CON lie within intervals that do not contain zero. For left amygdala-CON, the indirect pathway was stronger at low levels of parental acceptance [0.006 (CIs 0.003–0.009; 9% mediated)] compared to high levels [0.003 (CIs 0.001–0.005; 6% mediated)]. For right amygdala-CON, the indirect pathway was present at low levels [0.004 (CIs 0.001–0.006; 6% mediated)] but not high levels [0.001 (CIs −0.0001 to 0.003)].

**Table 3.** Moderation of pubertal timing and the family environment predicts corticolimbic connectivity

	Family conflict	Parental acceptance
LAmy-SMN-M	*B* = −0.000, s.e. = 0.001, *T* = −0.342, *p* = 0.732	*B* = 0.000, s.e. = 0.003, *T* = 0.312, *p* = 0.755
LAmy-CON	*B* = −0.000, s.e. = 0.001, *T* = −0.040, *p* = 0.970	*B* = 0.006, s.e. = 0.003, *T* = 2.469, *p* = 0.014
RAmy-CON	*B* = −0.000, s.e. = 0.001, *T* = −0.327, *p* = 0.744	*B* = 0.007, s.e. = 0.003, *T* = 2.419, *p* = 0.016
RHip-CON	*B* = −0.000, s.e. = 0.001, *T* = −0.657, *p* = 0.511	*B* = 0.007, s.e. = 0.002, *T* = 2.557, *p* = 0.011
LHip-VAN	*B* = 0.000, s.e. = 0.001, *T* = 0.487, *p* = 0.626	*B* = 0.000, s.e. = 0.003, *T* = 0.025, *p* = 0.98

LAmy, Left Amygdala; RAmy, Right Amygdala; LHip, Left Hippocampus; RHip, Right Hippocampus; CON, Cingulo-Opercular Network; SMN-M, Somatomotor Network – Mouth; VAN, Ventral Attention Network.

Statistics are presented for the interaction term between pubertal timing and family environment predicting connectivity.

## Discussion

The current study investigated the role of corticolimbic connectivity in mediating well-established associations between earlier pubertal timing and mental health problems. Findings highlighted decreased corticolimbic connectivity in adolescents undergoing puberty earlier than their peers, which mediated associations between earlier timing and withdrawn depressed problems. Further, a positive family environment characterized by parental acceptance was found to buffer against these risk patterns in amygdala-CON, as it was related to less reductions in connectivity in those with earlier pubertal timing.

There was a general decrease in corticolimbic connectivity in adolescents at later stages of puberty, particularly for higher order networks (i.e. CON, frontoparietal, default mode and salience networks). These findings extend prior literature (Thijssen et al., 2020; van Duijvenvoorde et al., 2019) to highlight widespread resting-state connectivity changes between limbic regions (specifically amygdala and hippocampus) and most cortical networks during puberty, reaching well beyond the prefrontal cortex. Findings are broadly similar to age-related changes that have been identified during this period (van Duijvenvoorde et al., 2019) which is expected given the strong correlation between age and pubertal stage. However, as there are inconsistencies in previously identified patterns of corticolimbic changes in prior literature, the current findings help to aide interpretation of our primary investigations into pubertal timing.

Beyond group-level changes during puberty, those who underwent puberty earlier than their peers exhibited greater (than average) reductions in bilateral amygdala-CON, right hippocampus-CON, left hippocampus-VAN, left hippocampus-VN and left amygdala-SMN-M connectivity. These findings particularly provide strong support for involvement of the CON, which is implicated in cognitive control processes such as maintenance of alertness and task-set over time (Menon & D'Esposito, 2022). Animal studies have reported high density of hormone receptors in the amygdala and hippocampus, as well as extensive anatomical connections between the amygdala and frontal and insular cortices (Aggleton, Burton, & Passingham, 1980; Ghashghaei, Hilgetag, & Barbas, 2007). Overall, the pattern of effects is suggestive of accelerated biological development in those who undergo puberty earlier than peers, when compared to connectivity changes identified for normative progression through pubertal stage. Indeed, both earlier pubertal timing and decreased corticolimbic connectivity have been independently considered markers of accelerated biological development, particularly within contexts of early life stress (Colich & McLaughlin, 2022). Earlier timing supports reproductive success in conditions of adversity and uncertainty (Belsky, 2019). Decreased connectivity (particularly amygdala-PFC) may also reflect accelerated maturation of emotional systems in response to external stressors [increasing top-down signaling through activation of higher-order cognitive control networks (Gee et al., 2013)]. Thus it is possible that stress-induced changes in the hypothalamic-pituitary-adrenal system and downstream changes in the hypothalamic-pituitary-gonadal system may underlie associations between early timing and greater-than-normative reductions in corticolimbic connectivity (Colich & McLaughlin, 2022).

There is a well-established relationship between early pubertal timing and mental health problems, particularly depression and conduct/delinquency problems (Ullsperger & Nikolas, 2017). It is often theorized that altered corticolimbic connectivity may mediate this association (Colich & McLaughlin, 2022), but few have empirically investigated this relationship. The current findings provide support for this model increasing risk for withdrawn depression, based on significant indirect pathways between earlier timing and mental health via decreased corticolimbic connectivity. In particular, effects involving the amygdala-CON were robust to the potential confounding effects of demographic characteristics (race/ethnicity and socioeconomic status). Findings broadly align with prior literature highlighting a potential role of accelerated corticolimbic connectivity during threat processing (Barendse et al., 2020; Spielberg et al., 2015) and similar patterns of white matter development (Kircanski et al., 2019) in predicting internalizing problems for those with advanced pubertal maturation. While the only prior study of resting-state functional connectivity failed to identify such pathways, it did not specifically focus on corticolimbic connections (Ernst et al., 2019). The prevalence of hormone receptors in the amygdala, and its white matter connections with the frontal and insular cortex, may place amygdala-CON connectivity as key risk pathways in those with earlier puberty. While decreased corticolimbic connectivity may reflect earlier maturation of emotional systems in response to early life stress, it may also decrease the window of neural plasticity to support maturation of emotional regulatory circuits and thus increase risk for depression (Callaghan & Tottenham, 2016).

Finally, there were significant interactions between the family environment and pubertal timing in predicting bilateral amygdala- and right hippocampus-CON connectivity; low levels of parental acceptance exacerbated the negative relationship between timing and connectivity, while high levels buffered against this pattern. Further, there was some support for contextual amplification models as parental acceptance moderated indirect pathways between pubertal timing and withdrawn depression via amygdala-CON connectivity, such that indirect pathways were not present at high levels of parental acceptance. This finding adds to the only prior study (to our knowledge) to examine and identify interactions between pubertal timing and parenting in relation to adolescent neural functioning (Barbosa et al., 2018). Individuals undergoing earlier puberty lean on their parents for guidance as they face the social challenges of this period (such as being physically different to their peers), and positive family environments are likely to be better equipped to support youth to navigate through these stressors (Rudolph & Troop-Gordon, 2010). Thus, to the extent that negative amygdala-CON connectivity reflects early activation of emotional circuits to respond to external stressors (Callaghan & Tottenham, 2016), we speculate that those in positive family environments may also receive external support with emotion regulation and may therefore require less reductions in connectivity (i.e. less acceleration of biological development) in response to socioemotional demands.

The current study represents one of the few empirical examinations of pathways between earlier pubertal timing, corticolimbic connectivity and mental health problems. It is also the first to specifically examine these relationships based on resting-state connectivity. However, an important limitation is that pubertal timing and stage are highly confounded, and thus is it difficult to tease apart neural changes that are specifically related to early timing. Nonetheless, we characterize patterns of normative neural changes as adolescents progress through the stages of puberty, and additional changes in those with earlier timing. Second, the availability of only two waves of assessments limits modeling of within-subject changes. As such, we used all available data across the two waves to examine between-subject effects (i.e. cross-sectional associations). This also limits inferences about causality based upon the mediation models. Future studies should examine additional waves of the ABCD to investigate longitudinal trajectories, as well as prospective mediation models. Third, there was a time lag (of approximately 1 year) between assessments of parental caregiving and other variables of interest for a subset of data points. Fourth, our analyses focused on one parcellation scheme used to centrally process rsfMRI data in ABCD, but it is important for future research to examine reproducibility of findings across different parcellation schemes. Fifth, there are many potential roles of the family environment in relation to puberty and corticolimbic connectivity. In addition to its modulation of pathways, there is support for negative family contexts being a sequelae and consequence of early pubertal timing. Indeed, prior research on the ABCD has identified indirect pathways between negative family environments and corticolimbic connectivity via earlier timing (Thijssen et al., 2020). Moreover, there are also several additional aspects of the family environment that co-occur with one another that should be interrogated in future studies to understand specificity to parental acceptance. Finally, future studies may also wish to interrogate whether similar associations are present when considering gender identity as opposed to biological sex.

In conclusion, the current investigation of the ABCD cohort highlights neural mechanisms that partially account for the increased risk for depression in adolescents experiencing puberty earlier than their peers. In particular, findings implicated decreased connectivity between limbic structures and the CON involved in higher-order cognitive control. Exploratory analyses provided preliminary support for the role of positive family environments in buffering against neural risk patterns (involving the amygdala) in adolescents undergoing earlier pubertal maturation. Conversely, negative family environments may exacerbate these neural risk pathways, and adolescents experiencing earlier puberty in such contexts represent key targets for interventions during the early adolescent years.

## Supporting information

Vijayakumar et al. supplementary material Vijayakumar et al. supplementary material

## Supplementary material

The supplementary material for this article can be found at https://doi.org/10.1017/S0033291723001472
